# Older adults with slow sit to stand times show reduced temporal precision of audio–visual integration

**DOI:** 10.1007/s00221-023-06628-3

**Published:** 2023-05-12

**Authors:** A. O’Dowd, R. J. Hirst, A. Setti, R. A. Kenny, F. N. Newell

**Affiliations:** 1 School of Psychology, Institute of Neuroscience, Trinity College Dublin, Dublin, Ireland; 2 The Irish Longitudinal Study on Ageing, Trinity College Dublin, Dublin, Ireland; 3 School of Applied Psychology, University College Cork, Cork, Ireland; 4 Mercer Institute for Successful Ageing, St James Hospital, Dublin, Ireland

**Keywords:** Ageing, Sit-to-stand, Multisensory, Sound induced flash illusion

## Abstract

Sustained integration of sensory inputs over increased temporal delays is associated with reduced cognitive and physical functioning in older adults and adverse outcomes such as falls. Here, we explored the relationship between multisensory integration and a clinically relevant measure of balance/postural control; Sit-to-Stand Time, the efficiency with which an older adult can transition between a seated and a standing posture. We investigated whether temporal multisensory integration was associated with performance on the Five-Times Sit-to-Stand Test (FTSST) in a large sample of 2556 older adults (mean age = 63.62 years, *SD* = 7.50; 55% female) drawn from The Irish Longitudinal Study on Ageing (TILDA). K-means clustering was applied to FTSST data, yielding three clusters characterised by fast (*mean* = 10.88 s; *n* = 1122), medium (*mean* = 14.34 s; *n* = 1133) and slow (*mean* = 18.97 s; *n* = 301) sit-to-stand times. At wave 3 of TILDA, older adults participated in the Sound Induced Flash Illusion (SIFI), a measure of the precision of temporal audio–visual integration, which included three audio–visual stimulus onset asynchronies (SOAs): 70, 150 and 230 ms. Older adults with the slowest sit-to-stand times were more susceptible to the SIFI at the longest SOA (230 ms) compared to the shortest SOA (70 ms) relative to those with the fastest times (*p* = 0.02). Older adults who take longer to repeatedly transition from a seated to a standing posture exhibit an expanded temporal binding window for audio–visual events, supporting a link between multisensory perception and balance/postural control in ageing.

**Supplementary Information:**

The online version contains supplementary material available at 10.1007/s00221-023-06628-3.

## Introduction

There is accumulating evidence that the temporal precision of multisensory integration changes with increasing age (Hernández et al. 2019; McGovern et al. 2014, 2022; Setti et al. 2011), whereby older adults show sustained integration of cross-sensory inputs separated over longer time delays than younger adults (Hernández et al. 2019; Hirst et al. 2020; McGovern et al. 2014, 2022; Setti et al. 2011). This age-related change to multisensory integration is potentially maladaptive as it is particularly evident in older adults with mild cognitive impairment (Chan et al. 2015) or with a history of falls (Setti et al. 2011). Associations between multisensory integration and balance function as well as postural control have also been reported in older adults (Mahoney et al. 2014, 2019; Merriman et al. 2015; Stapleton et al. 2014; Zhang et al. 2020). Maintaining stable balance and posture over any time duration relies on the processing and integration of information from multiple sensory modalities, including the visual, somatosensory, proprioceptive and vestibular systems (Bronstein 2016; Chiba et al. 2016). As such, changes in multisensory integration with increasing age may adversely affect these critical functions required for good mobility and possibly contribute to the occurrence of a fall incident (Mahoney et al. 2019; Setti et al. 2011; Zhang et al. 2020), which can have serious quality of life and potentially life-threatening consequences for an older adult (Ambrose et al. 2013). A more thorough understanding of the contributory factors of functional decline in ageing is necessary to assist in the prevention of such outcomes, given that fall incidents are generally difficult to predict (Donoghue et al. 2022), yet several risk factors are potentially modifiable (Ambrose et al. 2013). Although there is growing evidence linking multisensory integration with balance function in older adults (Zhang et al. 2020), this research has typically involved relatively small sample sizes, which is particularly challenging given the complexity of falls (Ambrose et al. 2013; Donoghue et al. 2022). Moreover, it remains unclear if the temporal dimension of multisensory integration specifically is associated with balance/postural control in older adults (Setti et al. 2011; Stapleton et al. 2014). This is important to clarify, given that the precision of temporal multisensory integration can be significantly improved with training in a relatively cost-effective and accessible manner (e.g., McGovern et al. 2022; Merriman et al. 2015; Setti et al. 2014).

In 2014, a measure of the precision of audio–visual temporal integration was integrated into a health assessment conducted at wave 3 of The Irish Longitudinal Study on Ageing (TILDA; Whelan and Savva 2013), which consists of a large sample (*N* = 8504) of older adults resident in the Republic of Ireland. To the best of our knowledge, TILDA remains the only large-scale study of ageing to incorporate a dedicated measure of multisensory integration into a comprehensive health assessment. This measure was the Sound-Induced Flash Illusion (SIFI; Shams et al. 2000, 2002), in which the presentation of a single ‘flash’ alongside two ‘beeps’ typically results in the perception of two ‘flashes’. The SIFI is widely considered as a valid and reliable measure of the temporal precision of audio–visual integration and, due to its relative simplicity, is appropriate for testing with older adults across a wide age range. Moreover, an increase in susceptibility to the SIFI at longer audio–visual temporal delays has been associated with the healthy ageing process (Hernández et al. 2019; McGovern et al. 2014, 2022; Setti et al. 2011), postural sway (Stapleton et al. 2014), a history of falls (Setti et al. 2011) and cognitive impairment (Chan et al. 2015), thus demonstrating the sensitivity of the SIFI to the cognitive and physical health of older adults. Indeed, empirical research involving the TILDA sample specifically has already yielded evidence for a relationship between less precise audio–visual integration and poorer global cognitive function (Hernández et al. 2019; Hirst et al. 2022), slower gait speed (Setti et al. 2023) and weaker grip strength (O’Dowd et al. in prep.).

Here, we focused on the Five-Times Sit-to-Stand Test (FTSST; Whitney et al. 2005), in which older adults transition from a seated to a standing posture five times in a row, without using their hands or arms for support. The FTSST is often utilised as a clinical measure of fall risk as it predominantly gauges lower limb strength, endurance, postural control and dynamic balance function (Goldberg et al. 2012; Lord et al. 2002; Meretta et al. 2006; Muñoz-Bermejo et al. 2021; Tiwari et al. 2019) and can discriminate those with balance disorders from their healthier counterparts (Whitney et al. 2005). Given this sensitivity to balance and postural functions (Goldberg et al. 2012; Lord et al. 2002; Meretta et al. 2006; Muñoz-Bermejo et al. 2021; Tiwari et al. 2019) and, as mentioned above, the integral role of multisensory processes in these functions (Bronstein 2016; Chiba et al. 2016; Mahoney et al. 2014, 2019; Merriman et al. 2015; Stapleton et al. 2014; Zhang et al. 2020), we hypothesised that performance on the FTSST would be associated with patterns of SIFI susceptibility in the TILDA sample. More specifically, we predicted that older adults exhibiting slower times on the FTSST would show increased susceptibility to the illusion at longer audio–visual delays (i.e., less precise patterns of multisensory integration) compared to their faster counterparts.

## Methods

### Study population

Participants were drawn from wave 3 (2014–2015) of TILDA, a population representative sample of 8504 individuals, resident in the Republic of Ireland (Whelan and Savva 2013). The study was approved by the Trinity College Dublin Faculty of Health Sciences Research Ethics Committee and complied with relevant data protection legislation. All participants provided informed consent at every testing wave. In total, 3968 older adults aged 50 years or over had available data for the Sound Induced Flash Illusion (SIFI) experiment, which was conducted only at wave 3. Consistent with previous studies involving the TILDA cohort, in which SIFI susceptibility was the outcome measure (Hernández et al. 2019; Hirst et al. 2022; O’Dowd et al. 2023; Setti et al. 2023), data were omitted prior to analysis for participants who met the following criteria: they were registered as legally blind (*n* = 2), had a suspected mild cognitive impairment (based on a Montreal Cognitive Assessment Score < 23; *n* = 456) and/or had missing/problematic data for important model predictors/covariates (*n* = 954, 433 of whom were missing sit-to-stand time data), including 21 older adults with self-reported levels of physical activity ≥ 16 h/day. This resulted in a final sample of 2556 older adults (mean age = 63.62 years, *SD* = 7.50; 55% female) whose data were available for analysis.

### Five-times sit-to-stand test

Older adults partook in the Five-Times Sit-to-Stand Test (FTSST) as part of a comprehensive health assessment at wave 3. They were asked to transition from a seated (chair height of 46 cm) to a standing posture five times in succession, without pause, as quickly as they could. For the entire duration of the test, both arms were folded across the chest. A trained healthcare nurse recorded the time taken to complete this test using a hand-held stopwatch. Timing commenced following a verbal prompt from the nurse (“*Are you ready? … Stand*”) and was terminated once the older adult had fully straightened at the end of the fifth stand. The nurse counted each stand aloud. Overall completion times varied from 5.84 to 33.91 s, with a group average of 13.37 s (*SD* = 2.98). As our aim was to investigate the association between performance on this test and multisensory integration, we applied k-means clustering using the ‘stats’ package in RStudio (Team R 2021) to the FTSST data, which were continuous in nature. The formation of clusters allowed us to retain the full range of values for the FTSST but mitigate the influence of particularly slow or fast times on the results. Moreover, this approach facilitated an examination of whether any association between FTSST performance and susceptibility to the SIFI was specific to a particular, potentially clinically significant, subgroup of older adults in the TILDA sample.

### Performance on the sound-induced flash illusion

Susceptibility to the Sound Induced Flash Illusion (SIFI; Shams et al. 2000, 2002), across three Stimulus Onset Asynchronies (SOAs), was included in the same healthcare assessment at wave 3 as a valid measure of the temporal precision of audio–visual integration (Hirst et al. 2020). The task was performed in the presence of a trained healthcare nurse. Participants were seated approximately 60 cm from a computer (Dell Latitude E6400 with Intel Core 2 Duo CPU, 2 Gb RAM, using Windows 7 Professional OS, 60 Hz refresh rate) and fixated on a fixation cross (1000 ms) located at the centre of the screen. On each trial, a visual stimulus (a white disc, 1.5° visual angle, approximately 32 fl luminance) and/or auditory [brief bursts 3500 Hz sounds (10 ms, 1 ms ramp)] stimuli were presented. The visual stimulus was presented on a black background (5 cm beneath the central fixation cross, approximately 4.7° visual angle), for 16 ms. The auditory stimuli were presented at approximately 80 dB over the computer speakers.

The main testing block contained a random order of multisensory illusory trials (2B1F, in which 2 Beeps are paired with 1 Flash), non-illusory trials (2B2F, 1B1F) and unisensory visual trials (0B2F, 0B1F), each presented twice. The participants reported the number of perceived visual flashes. Unisensory auditory trials (2B0F, 1B0F) were presented in a separate block and participants reported the number of perceived auditory beeps. Participants’ vocal responses were recorded by the nurse who pressed the corresponding number key on a laptop. Illusion trials of the SIFI consisted of three SOAs, 70, 150 and 230 ms, and the second beep either preceded (pre) or followed (post) the flash-beep pair. Due to time constraints within the overall TILDA protocol, a total of 12 ‘illusory’ trials were completed (i.e., two trials per SOA across pre–post conditions). Overall, the SIFI was completed in approximately 6 min, which was the maximum length of time allocated to the experiment during the health assessment.

### Analysis

The main outcome of this study was response accuracy to the illusion trials (2 beeps with 1 flash) of the SIFI. An accurate response indicated that the participant was not susceptible to the illusion on that trial. As there were two trials per condition of the SIFI within TILDA, accuracy took the form of 0 (i.e., an incorrect response on both trials, indicating consistent illusion susceptibility), 0.5 (correct response on one trial) or 1 (correct response on both trials) and was treated as a discrete variable. Given that these data were discrete and the SIFI task was within-subjects in nature (i.e., every participant was exposed to three audio–visual stimulus onset asynchronies for both the pre- and post-conditions), generalised logistic mixed effects regression models were fitted via the ‘lme4’ package (Bates et al. 2014). This approach not only allowed for a robust analysis of these data by facilitating the explicit modelling of random effects as sources of variability as well as the inclusion of multiple categorical and continuous covariates but also ensured that the assumption of independence for a within-subjects design was not violated.

The following terms were included in the model: age, stimulus onset asynchrony (SOA; 70, 150, 230 ms), sit-to-stand cluster (STScluster; slow, medium, fast), sex (male, female), education (primary, secondary, tertiary), self-reported vision and audition (poor to excellent), visual acuity score (VAS = 100 − 50*LogMAR), hearing aid use (yes, no), number of cardiac (stroke, ministroke, heart attack, heart murmur, abnormal heart rhythm and/or angina; 0, 1, 2 +) and non-cardiac (Parkinson’s Disease, chronic lung disease, asthma, arthritis, osteoporosis and/or cancer; 0, 1, 2 +) diseases, depression (CES-D ≥ 9), weekly physical activity (metabolic minutes a week based on the International Physical Activity Questionnaire—Short Form), body mass index (BMI; kg/m^2^), accuracy (0, 0.5, 1) on multisensory congruent (1 beep and 1 flash), unimodal visual (2 flashes only; 70 ms SOA) and unimodal auditory trials (2 beeps only; 70 ms SOA) and pre–post condition. As our aim was to examine whether there was a genuinely unique association between the FTSST and multisensory integration, the models were also adjusted for MoCA score, grip strength and performance on the Timed Up and Go task (TUG), to control for global cognition, body strength and functional mobility. The analysis model also included SOA*age, SOA*sex, SOA*MoCA, SOA*TUG, SOA*Grip and SOA*pre–post interaction terms and a random intercept term (participant ID). To address our hypothesis that performance on the FTSST test would be associated with the precision of temporal multisensory integration in older adults, we examined whether the effect of SOA on accuracy, using the 70 ms as the reference condition, interacted with the STS cluster (SOA*STScluster). The statistical significance of the SOA*STScluster interaction term was determined via a likelihood ratio test, where we evaluated the fit of the model with and without this interaction term while holding constant all of the aforementioned covariates.

The analysis was conducted with R in R studio (Team R 2021). Supplementary materials (full model results and R script) are available at https://osf.io/75mur/?view_only=f558a2b2e72648e0909ed4ce5d1370eb. All continuous variables were scaled prior to analysis.

## Results

### FTSST clusters

**Fig. 1:**
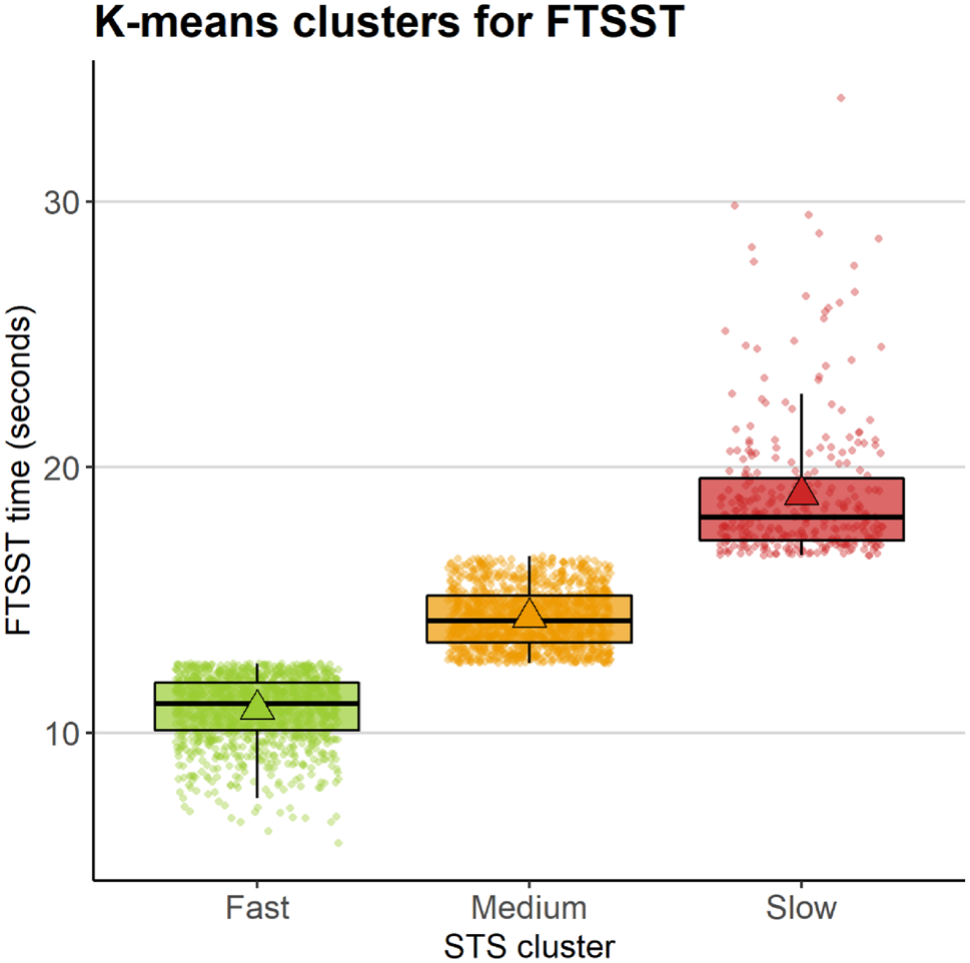
Plots show the clusters for average Five-Times Sit-to-Stand Test (FTSST) times. Boxplots show the distribution of the data for each cluster. Circles show individual data points and large triangles show the mean values per cluster

K-means clustering was applied to the FTSST data for the full analysis sample (*N* = 2556) using 10 maximum iterations and 100 starting positions, with 2–5 cluster solutions tested. Cross-sectional k-means clustering produced an optimal solution of three clusters for sit-to-stand times. This solution was identified as optimal via the ‘nbClust’ package (Charrad et al. 2014) in R studio based on the majority rule and the Caliński–Harabasz (Caliński and Harabasz 1974) criterion, a well-established method of determining the optimal clustering solution. The final clusters are shown in Fig. 1. The cluster centres were as follows: 10.88 s (fast; *n* = 1122; *SD* = 1.25), 14.34 s (medium; *n* = 1133; *SD* = 1.09) and 18.97 s (slow; *n* = 301; *SD* = 2.66).

### Multisensory integration (SIFI performance)

**Fig. 2:**
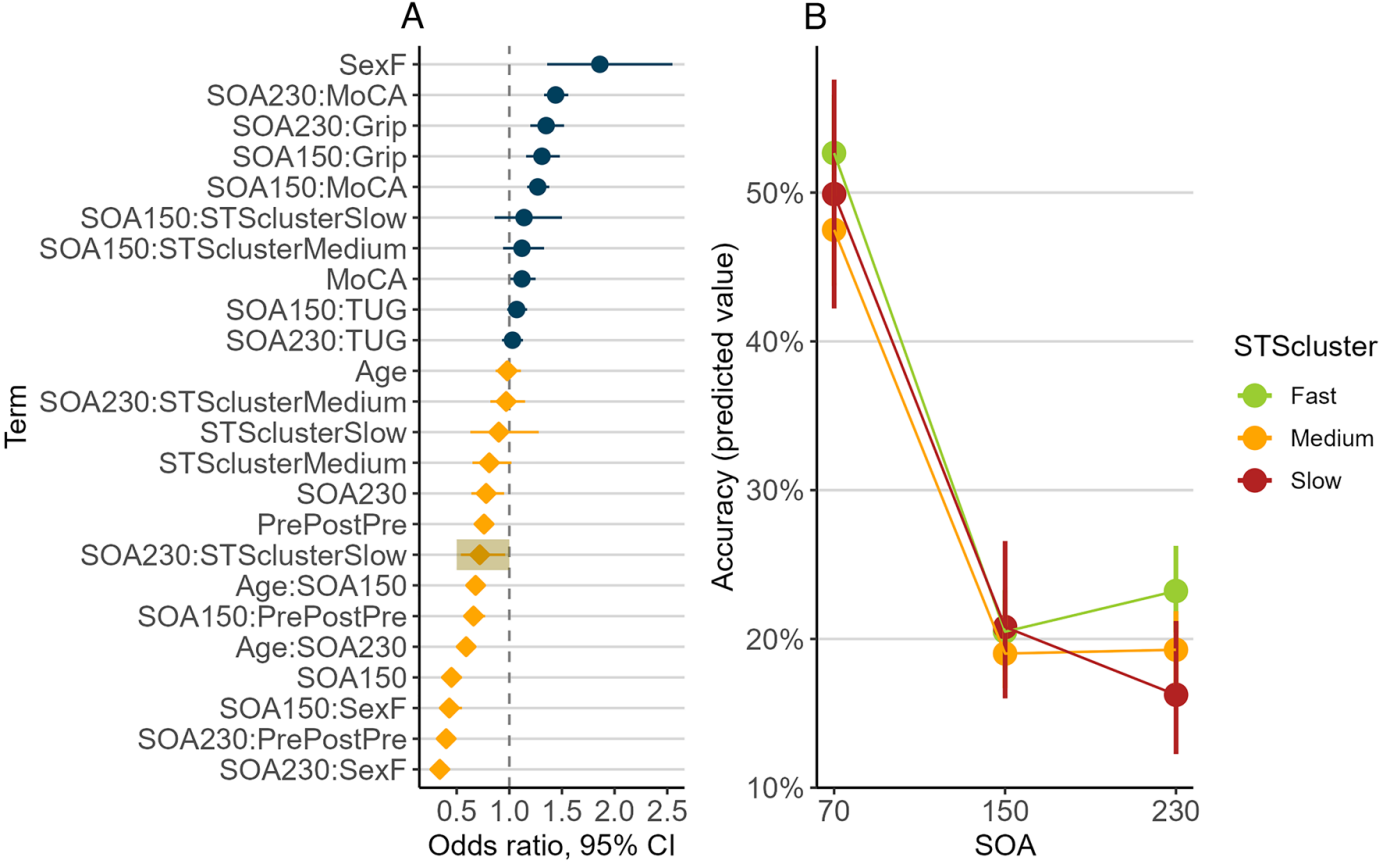
Summary of the results of generalised logistic mixed-effects regression models predicting accuracy on 2B1F trials of the SIFI. **A** Plot shows the odds ratios for making a correct response on illusory (2B1F) SIFI trials (with 95% confidence intervals). Odds ratios to the left of the vertical dashed line (yellow diamonds) indicate reduced odds of making a correct response (i.e., increased illusion susceptibility) and odds ratios to the right of the vertical dashed line (blue circles) indicate increased odds of making a correct response (i.e., reduced illusion susceptibility). The odds ratio for the statistically significant interaction between SOA and sit-to-stand cluster (STScluster) is highlighted. **B** The predicted accuracy values (marginal means) for the illusion (2B1F) trials of the SIFI for all STS clusters. Higher values indicate increased accuracy in identifying the number of flashes presented (whilst ignoring the beeps), therefore reduced susceptibility to the illusion. Error bars indicate 95% confidence intervals

The likelihood ratio test revealed that the SOA*STScluster term significantly contributed to the model predicting accuracy on illusion trials of the SIFI (χ^2^_(2)_ = 11.64, *p* = 0.02) as shown in Fig. 2A. This interaction was driven by increased SIFI susceptibility at the 230 ms SOAs relative to the 70 ms SOA for those in the slow STScluster compared to those in the fast STScluster, as shown in Fig. 2B. More specifically, older adults with the slowest STS times exhibited 28% lower odds of making an accurate response at 230 ms (odds ratio = 0.72, 95% CI [0.54, 0.96]), relative to 70 ms, compared to older adults with the fastest STS times. As shown in Fig. 2B and Table 1, the difference in the predicted accuracy value (marginal means) was 30% vs 34% for the fastest and slowest STS clusters respectively at 230 ms (vs 70 ms). Full model results are available in Supplementary Materials, Table S2.

**Table 1:** The predicted accuracy value [marginal means; 95% confidence intervals] across stimulus onset asynchronies (SOA; 70, 150, 230 ms) for each of the STS clusters (slow, medium, fast)

	70 ms	150 ms	230 ms	70 vs 150	70 vs 230
Fast STS (*n* = 1122)	53% [49,57]	20% [18,23]	23% [20,26]	33%	30%
Medium STS (*n* = 1133)	47% [44,51]	19% [17,22]	19% [17,22]	28%	28%
Slow STS (*n* = 301)	50% [42,58]	21% [16,27]	16% [12,21]	29%	34%

## Discussion

We investigated the relationship between performance on the Five-Times Sit-to-Stand Test (FTSST), a clinical measure of lower limb strength, endurance and balance/postural function, and susceptibility to the Sound Induced Flash Illusion (SIFI; Shams et al. 2000, 2002), which assesses the precision of temporal audio–visual integration. Our results revealed that, among a large sample (*N* = 2556) of community-dwelling older adults, sit-to-stand (STS) performance was significantly associated with patterns of audio–visual integration. More specifically, the size of the difference between performance at the shortest (70 ms) vs longest (230 ms) stimulus onset asynchrony (SOA) of the SIFI was larger in a group of older adults with the slowest STS times compared to those with the fastest STS times. Although the size of this difference in predicted values (marginal means) between the slowest and fastest groups was relatively small (4%), increased illusion susceptibility at the longest audio–visual delay does indicate reduced temporal precision of multisensory integration (Hernández et al. 2019; Hirst et al. 2020; McGovern et al. 2014, 2022; Setti et al. 2011). Therefore, older adults who are relatively slow to repeatedly transition from a seated to a standing posture exhibit an expanded audio–visual temporal binding window (TBW), the window in which auditory and visual signals are most likely to be integrated (Diederich and Colonius 2013; Hernández et al. 2019; Hirst et al. 2020; McGovern et al. 2014, 2022; Meredith et al. 1987; Setti et al. 2011). Of note, there was no group difference in overall susceptibility to the SIFI in this sample, emphasising the importance of incorporating SOA as a factor when using the SIFI measure. This is consistent with other evidence from the TILDA sample that increased SIFI susceptibility at longer versus shorter SOAs is associated with reduced cognitive function (Hernández et al. 2019; Hirst et al. 2022), slower gait speed (Setti et al. 2023) and weaker grip strength (O’Dowd et al., in prep.).

To account for this group difference in SIFI susceptibility across SOAs, we suggest a possible link between the temporal precision of multisensory integration and postural/balance control in older adults. An individual’s ability to maintain and control their sense of balance and posture is known to become more challenging with increasing age (Choy et al. 2003; Era et al. 2006), in part because the sensory systems experience a degree of natural decline (Anson et al. 2017). As described in the introduction, the processing of signals across multiple sensory modalities is intrinsic to the maintenance of balance and posture (Bronstein 2016; Chiba et al. 2016). In this respect, the ability to efficiently integrate sensory signals is important as is the appropriate weighting of these inputs (Assländer and Peterka 2014; Bronstein 2016; Chiba et al. 2016; Mahboobin et al. 2009; Oie et al. 2002); that is, the influence of different sensory modalities on postural control should be determined based on their relative reliability and sensory contributions to postural control appear to be different in younger versus older adults (e.g., Wiesmeier et al. 2015). Perhaps unsurprisingly, therefore, a link between balance performance (e.g., unipedal stance time) and multisensory integration has been reported in older adults (Zhang et al. 2020), including evidence that patterns of multisensory integration are predictive of balance function (Mahoney et al. 2019). Moreover, a wider TBW appears to coincide with reduced vestibular function (Chang et al. 2012; Shayman et al. 2018) and fall-proneness (Setti et al. 2011; Zhang et al. 2020) while SIFI susceptibility is greater in fall-prone older adults during a standing relative to a sitting position (Stapleton et al. 2014). It has been suggested that a widened TBW could influence balance/posture control directly, through changes in these sensory integration and/or reweighting processes, or indirectly, by promoting increased processing of irrelevant stimuli and consequentially limiting resources allocated to balance/postural control (Setti et al. 2011). Importantly, performance on the FTSST is sensitive to posture/balance function in older adults, given that the task involves rapidly and repeatedly transitioning from a seated to a standing position without any assistance from the arms or hands (Goldberg et al. 2012; Lord et al. 2002; Meretta et al. 2006; Muñoz-Bermejo et al. 2021; Tiwari et al. 2019; Whitney et al. 2005). Moreover, the percentage of older adults who reported feeling unsteady or only slightly steady while standing, walking and rising from a chair was highest in the slowest STS group (see Supplementary Materials), which would appear to reinforce an association between less precise multisensory integration and broader differences in balance/postural stability specifically in ageing. While the FTSST can also be influenced by cognition, body strength and mobility (Annweiler et al. 2011; Goldberg et al. 2012; Lord et al. 2002; Meretta et al. 2006; Muñoz-Bermejo et al. 2021; Tiwari et al. 2019; Whitney et al. 2005), we adjusted our statistical models for MoCA scores, grip strength (one the dominant hand) and TUG times to control for possible contributions from global cognition, strength and functional mobility respectively to our results. Therefore, we propose that these factors are unlikely to, at least fully, account for our findings. Indeed, there was no evidence for a significant interaction between SOA and TUG times recorded at wave 3, consistent with evidence from our research group that longitudinal (10-year) TUG performance is also not associated with SIFI susceptibility in the TILDA sample (O’Dowd et al. 2023). The reasons for such remain unclear, given that the TUG task also comprises a chair stand and assesses dynamic balance (Herman et al. 2011). It may be that that the act of repeatedly transitioning between postures as quickly as possible and without the support of the upper limbs was more challenging to postural control for these older adults than the TUG task—for example, the chair stand during the TUG can be assisted by the upper limbs, the task can be completed with the use of a walking aid and participants are allowed to take breaks if necessary, factors which do not apply to the FTSST. Nevertheless, this is speculative and we recognise that the absence of any standalone, objective measure of balance/postural function specifically in the TILDA project is a limitation regarding the interpretation of our results. However, there is evidence that performance on the TUG task is associated with patterns of visuo-tactile integration in community-dwelling older adults (Hide et al. 2021). As such, further empirical research is needed to ascertain whether clinically validated measures of balance/postural control are differentially sensitive to performance on certain tasks of multisensory integration or to particular combinations of sensory modalities. This would help to shed light on the optimal approach to take when investigating the relationship between multisensory integration and inefficient balance/postural function as well as falls, a major public health issue, in ageing populations. Indeed, we acknowledge that the current study involved a measure of temporal audio–visual integration only. Other studies have suggested a relationship between balance/postural function and the integration of inputs from other sensory modalities more intrinsic to the body itself, such as vision, touch and proprioception (Mahoney et al. 2014, 2019; Zhang et al. 2020), suggesting that patterns of multisensory integration more broadly are associated with balance/postural control as well as falls in older adults. Future work could incorporate both the SIFI and a variant of this task, the touch-induced visual illusion (e.g., Violentyev et al. 2005), to directly compare how the temporal precision of multisensory integration for different sensory pairings is associated with performance on measures of balance and postural control in older adults.

That the observed group difference in SIFI susceptibility was specific to those in the slowest versus fastest STS groups is potentially of clinical significance. The intermediate STS group exhibited an STS time that was very close to the overall group average (14.3 and 13.4 s respectively) and they performed comparably to those in the fastest group on the SIFI. As such, only a specific subgroup of older adults, who exhibited STS times consistently above 16 s (mean = 18.97 s, range of 16.7–33.9 s), exhibited significantly less precise patterns of temporal audio–visual integration. Interestingly, previous empirical research involving the TILDA sample has suggested a cut-off of 15 s for probable sarcopenia (Duggan et al. 2022), a condition typified by a loss of skeletal muscle mass, muscle strength and muscle quality (Duggan et al. 2022; Kim et al. 2020). Sarcopenia can be associated with postural dysfunction and balance disorders (Gadelha et al. 2018; Kim et al. 2020; Muhlberg and Sieber 2004), likely reflecting the critical contribution of lower limb muscle strength to postural stability (Horlings et al. 2008; Orr 2010). As such, it is possible that our results are reflective of an expanded TBW in older adults with particularly pronounced age-related weakening of the lower limbs and associated difficulties with balance/postural control. However, further empirical research is needed to investigate whether temporal multisensory integration is particularly imprecise in *confirmed* cases of sarcopenia. This is particularly important to establish given evidence that sarcopenia can also be characterised by reduced cognitive function (Scisciola et al. 2021).

Whether there is a causal relationship between performance on the FTSST and multisensory processes cannot be readily determined based on our results, given that both measures are only cross-sectionally available in the TILDA study. Maintaining balance and posture as well as integrating sensory signals in time are core functions of the brain (Sousa et al. 2012; Surgent et al. 2019; Wise and Barnett-Cowan 2018). Therefore, the present findings could reflect an influence of reduced integrity of the structure and/or function of the brain in a broad sense among a specific subgroup of older adults in this sample. This could stem from a core causal mechanism(s), such as reduced white matter integrity or reduced processing speed, in line with the ‘common-cause hypothesis’ of ageing (Christensen et al. 2001; Li and Lindenberger 2002; Lindenberger and Ghisletta 2009). Indeed, the healthy ageing process is known to result in broad changes to the structural and functional characteristics of the brain (Bethlehem et al. 2022; Satoh et al. 2017) and white matter integrity is associated with postural control in older adults in response to changes in sensory feedback (Van Impe et al. 2012). However, longitudinal investigations of the precision of multisensory integration are needed to establish the precise nature of the relationship between multisensory perception and cognitive/physical health over time in older adults, including the directionality and potential causality of the relationships between these complex, interrelated domains of functioning. Grey matter volume in the right angular gyrus is associated with patterns of SIFI susceptibility at longer SOAs in the TILDA sample (Hirst et al. 2021). The angular gyrus has been described as a ‘convergence zone’, linked with multiple functions (Seghier 2013), including sensory integration, higher-order motor control (Farrer et al. 2008) and body representation (Spitoni et al. 2013). Critically, its extensive connections with multiple cortical and subcortical brain regions has suggested a role of the angular gyrus in sensory integration for motor planning and motor control (Petit et al. 2023). As such, the right angular gyrus is a candidate region for future neuroimaging studies investigating the relationship between multisensory integration and balance/postural functions in older adults. The present finding for a significant link between audio–visual temporal integration and the time taken to complete the FTSST further reinforces that precision in multisensory perception is an important dimension of the ageing process, sensitive to the efficiency with which an older adults can transition between a standing and a seated posture. Further establishing the nature of this relationship is important given that both the sit-to-stand performance and the temporal precision of multisensory integration are independently associated with balance and falls in older adults (Goldberg et al. 2012; Lord et al. 2002; Mahoney et al. 2019; Setti et al. 2011; Tiwari et al. 2019; Whitney et al. 2005; Zhang et al. 2020) and both are potentially modifiable (McGovern et al. 2022; Merriman et al. 2015; Schot et al. 2003; Setti et al. 2014). As such, we propose that multisensory integration, which can be assessed in an accessible and cost-effective manner, should be afforded greater attention in scientific studies of the ageing brain and healthcare assessments as a valuable indictor of health in ageing.

## Conclusions

We investigated the relationship between the temporal precision of multisensory integration and performance on the Five-Times Sit-To-Stand Test (FTSST) in a large sample of community-dwelling older adults (*N* = 2556) drawn from The Irish Longitudinal Study on Ageing (TILDA). A group of older adults who showed an average sit-to-stand time of 18.97 s (slowest) were more susceptible to the Sound Induced Flash Illusion (SIFI) at an extended audio–visual delay compared to those with an average time of 10.88 s (fastest). An intermediate group, who showed an average sit-to-stand time of 14.34 s, showed no difference in SIFI susceptibility at this delay compared to the fastest group. Our results suggest that less precise temporal integration is evident in a specific group of older adults who are particularly slow at repeatedly transitioning from a seated to a standing posture. We propose that our findings reflect a relationship between the temporal precision of multisensory integration and balance/postural control in older adults.

## Supplementary Information

Below is the link to the electronic supplementary material.

Supplementary file1 (DOCX 33 KB)

## Data Availability

To acquire access to the data from The Irish Longitudinal Study on Ageing (TILDA), see https://tilda.tcd.ie/data/accessing-data/. Data from the Sound Induced Flash Illusion are available in the publicly released dataset which can be accessed via a hotdesk facility or by requesting access through the Irish Social Science Data Archive or the Interuniversity Consortium for Political and Social Research. Information on how to access these data as well as direct links to the request forms can be found at https://tilda.tcd.ie/data/accessing-data/ and https://tilda.tcd.ie/data/accessing-data/hotdesk/.
