# Supplementary material for: Older adults with slow sit to stand times show reduced temporal precision of audio–visual integration
Source: Exp Brain Res. 2023 May 12;241(6):1633–42. doi: 10.1007/s00221-023-06628-3 (PMC10224838; doi:10.1007/s00221-023-06628-3)
Supplement: Supplementary file 1 — Supplementary file1 (DOCX 33 KB) [file 221_2023_6628_MOESM1_ESM.docx]

**Older adults with slow sit to stand times show reduced temporal precision of audio-visual integration**

**O’ Dowd^1,2^, A., Hirst^1,2^, R. J., Setti^2,3^, A., Kenny^2,4^, R .A., & Newell^1^, F. N.**

^1^School of Psychology and Institute of Neuroscience, Trinity College Dublin

^2^The Irish Longitudinal Study on Ageing, Trinity College Dublin

^3^School of Applied Psychology, University College Cork

^4^Mercer Institute for Successful Ageing, St James. Hospital, Dublin

**Corresponding author:** Alan O’ Dowd, School of Psychology and Institute of Neuroscience, Trinity College Dublin, Dublin, Ireland. Email: odowda1@tcd.ie

**Supplementary Materials**

| **Table S1.** Descriptive statistics for numerical (mean, SD) and categorical (count, %) variables across the three sit-to-stand time clusters (Fast, Medium, Slow). | | | | |
| --- | --- | --- | --- | --- |
| **Numerical, mean (SD)** | **Fast**  **(*N* = 1,122)** | **Medium**  **(*N* = 1,133)** | **Slow**  **(*N* = 301)** | ***p*** |
| Age, yrs | 62.09 (6.84) | 64.23 (7.70) | 67.04 (7.59) | **<.001** |
| Sit-to-stand time (s) | 10.88 (1.25) | 14.34 (1.09) | 18.97 (2.66) | **<.001** |
| Timed Up and Go (s) | 8.15 (1.93) | 8.99 (1.52) | 10.72 (4.13) | **<.001** |
| Gait speed (cm/s)^a^ | 147 (16.4) | 137 (16.5) | 123 (17.6) | **<.001** |
| Dominant hand grip strength (kg) | 29.87 (9.68) | 26.86 (9.14) | 25.36 (9.22) | **<.001** |
| Visual acuity (VAS) | 96.59 (8.22) | 96.37 (8.73) | 95.27 (7.67) | .052 |
| Global cognition (MoCA) | 27.04 (2) | 26.91 (2.02) | 26.59 (2.03) | **.002** |
| Body mass index (kg/m^2^) | 27.91 (4.53) | 28.05 (4.38) | 28.67 (4.82) | **.04** |
| Weekly exercise (METs)^b^ | 3,975 (5,284) | 3,123 (4,577) | 2,461 (3,860) | **<.001** |
| **Categorical *n* (%)** |  |  |  |  |
| Sex – female^b^ | 575 (51) | 651 (57) | 167 (55) | **.01** |
| Education – tertiary^b^ | 541 (48) | 503 (44) | 142 (47) | .40 |
| Fair/poor vision^b^ | 68 (6) | 62 (5) | 26 (9) | .12 |
| Fair/poor hearing^b^ | 158 (14) | 133 (12) | 47 (16) | .11 |
| Hearing aid user^b^ | 49 (4) | 66 (6) | 29 (10) | **.002** |
| Cardiac disease^b^ | 37 (3) | 48 (4) | 20 (7) | **.03** |
| Noncardiac disease^b^ | 110 (10) | 118 (10) | 36 (12) | .55 |
| Depression (CESD ≥ 9) | 78 (7) | 78 (7) | 35 (12) | **.01** |
| Faller – yes^b^ | 200 (18) | 240 (21) | 66 (22) | .18 |
| Fear of falling – yes^b^ | 173 (15) | 235 (21) | 86 (29) | **<.001** |
| Unsteady walking^b^ | 66 (6) | 149 (13) | 76 (25) | **<.001** |
| Unsteady standing^b^ | 46 (4) | 114 (10) | 53 (18) | **<.001** |
| Unsteady rising from a chair^b^ | 122 (11) | 210 (19) | 82 (27) | **<.001** |
| ^a^ *n* = 25 missing data for gait speed.  ^b^Self-reported. | | | | |

| **Table S2.** Full model results. | | | | | | | | | |
| --- | --- | --- | --- | --- | --- | --- | --- | --- | --- |
| **Term** | ***β*** | **Std. error** | **Lower 95% CI** | **Upper 95% CI** | ***z*** | **Odds ratio** | ***p*** | **Lower 95% CI** | **Upper 95% CI** |
| (Intercept) | -3.57 | 1 | -5.52 | -1.62 | -3.59 | 0.03 | <.001 | 0 | 0.2 |
| Age | -0.02 | 0.06 | -0.14 | 0.1 | -0.31 | 0.98 | 0.75 | 0.87 | 1.1 |
| SOA[150] | -0.8 | 0.1 | -0.99 | -0.6 | -7.99 | 0.45 | <.001 | 0.37 | 0.55 |
| SOA[230] | -0.25 | 0.1 | -0.44 | -0.05 | -2.46 | 0.78 | 0.01 | 0.64 | 0.95 |
| Sex[Female] | 0.62 | 0.16 | 0.31 | 0.93 | 3.88 | 1.86 | <.001 | 1.36 | 2.54 |
| PrePost[Pre] | -0.28 | 0.05 | -0.38 | -0.18 | -5.28 | 0.76 | <.001 | 0.68 | 0.84 |
| Education[Secondary] | 0.13 | 0.15 | -0.17 | 0.43 | 0.88 | 1.14 | 0.38 | 0.85 | 1.54 |
| Education[Tertiary] | 0.32 | 0.15 | 0.02 | 0.63 | 2.11 | 1.38 | 0.04 | 1.02 | 1.87 |
| MoCA | 0.11 | 0.05 | 0.01 | 0.22 | 2.07 | 1.12 | 0.04 | 1.01 | 1.24 |
| BMI | 0.02 | 0.05 | -0.07 | 0.11 | 0.41 | 1.02 | 0.68 | 0.93 | 1.12 |
| MET | -0.09 | 0.05 | -0.18 | 0.01 | -1.76 | 0.92 | 0.08 | 0.83 | 1.01 |
| STScluster[Medium] | -0.21 | 0.11 | -0.43 | 0.01 | -1.83 | 0.81 | 0.08 | 0.65 | 1.01 |
| STScluster[Slow] | -0.11 | 0.18 | -0.47 | 0.25 | -0.61 | 0.89 | 0.54 | 0.63 | 1.28 |
| Grip | -0.07 | 0.08 | -0.23 | 0.09 | -0.88 | 0.93 | 0.38 | 0.79 | 1.09 |
| TUG | -0.05 | 0.06 | -0.16 | 0.07 | -0.79 | 0.95 | 0.43 | 0.85 | 1.07 |
| VAS | -0.04 | 0.05 | -0.14 | 0.06 | -0.74 | 0.96 | 0.46 | 0.87 | 1.06 |
| SR_vision[Fair] | -0.45 | 0.71 | -1.83 | 0.94 | -0.63 | 0.64 | 0.53 | 0.16 | 2.57 |
| SR_vision[Good] | -0.71 | 0.69 | -2.05 | 0.63 | -1.04 | 0.49 | 0.3 | 0.13 | 1.88 |
| SR_vision[Very Good] | -0.64 | 0.69 | -1.99 | 0.7 | -0.94 | 0.52 | 0.35 | 0.14 | 2.02 |
| SR_vision[Excellent] | -0.5 | 0.69 | -1.86 | 0.85 | -0.73 | 0.6 | 0.47 | 0.16 | 2.35 |
| SR_audition[Fair] | -0.03 | 0.43 | -0.86 | 0.81 | -0.07 | 0.97 | 0.95 | 0.42 | 2.24 |
| SR_audition[Good] | -0.05 | 0.41 | -0.86 | 0.76 | -0.12 | 0.95 | 0.91 | 0.42 | 2.14 |
| SR_audition[Very Good] | -0.19 | 0.42 | -1 | 0.63 | -0.45 | 0.83 | 0.65 | 0.37 | 1.87 |
| SR_audition[Excellent] | -0.21 | 0.42 | -1.03 | 0.62 | -0.49 | 0.81 | 0.63 | 0.36 | 1.86 |
| HearingAid[Yes] | 0.86 | 0.21 | 0.44 | 1.28 | 4.03 | 2.37 | <.001 | 1.56 | 3.6 |
| Cardiac[1] | 0.08 | 0.25 | -0.41 | 0.58 | 0.33 | 1.09 | 0.74 | 0.66 | 1.78 |
| Cardiac[2+] | 0.52 | 0.76 | -0.97 | 2.02 | 0.68 | 1.69 | 0.49 | 0.38 | 7.52 |
| NonCardiac[1] | 0.13 | 0.16 | -0.19 | 0.44 | 0.8 | 1.14 | 0.43 | 0.83 | 1.55 |
| NonCardiac[2+] | -0.16 | 0.55 | -1.25 | 0.93 | -0.29 | 0.85 | 0.77 | 0.29 | 2.52 |
| Depression[Yes] | -0.12 | 0.18 | -0.48 | 0.24 | -0.67 | 0.88 | 0.50 | 0.62 | 1.27 |
| Cong_1B1F[0.5] | 3.42 | 0.64 | 2.16 | 4.68 | 5.32 | 30.62 | <.001 | 8.68 | 107.94 |
| Cong_1B1F[1] | 4.31 | 0.59 | 3.15 | 5.46 | 7.32 | 74.16 | <.001 | 23.42 | 234.86 |
| Uni_0B2F[0.5] | -0.59 | 0.12 | -0.82 | -0.36 | -5.11 | 0.55 | <.001 | 0.44 | 0.69 |
| Uni_0B2F[1] | -0.71 | 0.16 | -1.03 | -0.4 | -4.43 | 0.49 | <.001 | 0.36 | 0.67 |
| Uni_2B0F[0.5] | -0.19 | 0.13 | -0.44 | 0.06 | -1.48 | 0.83 | 0.14 | 0.64 | 1.06 |
| Uni_2B0F[1] | -0.01 | 0.11 | -0.23 | 0.21 | -0.09 | 0.99 | 0.93 | 0.79 | 1.23 |
| Age:SOA[150] | -0.38 | 0.05 | -0.47 | -0.29 | -8.34 | 0.68 | <.001 | 0.62 | 0.75 |
| Age:SOA[230] | -0.53 | 0.05 | -0.62 | -0.44 | -11.38 | 0.59 | <.001 | 0.54 | 0.64 |
| SOA150:Sex[Female] | -0.84 | 0.12 | -1.08 | -0.61 | -6.92 | 0.43 | <.001 | 0.34 | 0.55 |
| SOA230:Sex[Female] | -1.09 | 0.12 | -1.33 | -0.85 | -8.88 | 0.34 | <.001 | 0.26 | 0.43 |
| SOA150:PrePost[Pre] | -0.41 | 0.08 | -0.56 | -0.26 | -5.29 | 0.66 | <.001 | 0.57 | 0.77 |
| SOA230:PrePost[Pre] | -0.93 | 0.08 | -1.08 | -0.77 | -11.71 | 0.4 | <.001 | 0.34 | 0.46 |
| SOA150:MoCA | 0.24 | 0.04 | 0.16 | 0.32 | 5.89 | 1.27 | <.001 | 1.17 | 1.38 |
| SOA230:MoCA | 0.37 | 0.04 | 0.28 | 0.45 | 8.84 | 1.44 | <.001 | 1.33 | 1.56 |
| SOA150:STScluster[Medium] | 0.11 | 0.09 | -0.06 | 0.28 | 1.31 | 1.12 | 0.19 | 0.94 | 1.33 |
| SOA230:STScluster[Medium] | -0.03 | 0.09 | -0.2 | 0.14 | -0.34 | 0.97 | 0.73 | 0.82 | 1.15 |
| SOA150:STScluster[Slow] | 0.13 | 0.14 | -0.15 | 0.41 | 0.91 | 1.14 | 0.36 | 0.86 | 1.5 |
| SOA230:STScluster[Slow] | -0.33 | 0.15 | -0.62 | -0.05 | -2.27 | 0.72 | 0.02 | 0.54 | 0.96 |
| SOA150:Grip | 0.27 | 0.06 | 0.15 | 0.39 | 4.37 | 1.31 | <.001 | 1.16 | 1.48 |
| SOA230:Grip | 0.3 | 0.06 | 0.18 | 0.42 | 4.86 | 1.35 | <.001 | 1.2 | 1.52 |
| SOA150:TUG | 0.07 | 0.04 | -0.02 | 0.16 | 1.55 | 1.07 | 0.12 | 0.98 | 1.17 |
| SOA230:TUG | 0.03 | 0.05 | -0.07 | 0.12 | 0.53 | 1.03 | 0.6 | 0.93 | 1.13 |
